# High-Frequency Transcranial Random Noise Stimulation for Auditory Hallucinations of Schizophrenia: A Case Series

**DOI:** 10.3390/biomedicines10112698

**Published:** 2022-10-25

**Authors:** Marine Mondino, Delphine Janin, Filipe Galvao, Jérôme Brunelin

**Affiliations:** 1 Pôle Est, Centre Hospitalier Le Vinatier, F-69500 Bron, France; 2 PSYR2 Team, Lyon Neuroscience Research Center, INSERM U1028, CNRS UMR5292, F-69000 Lyon, France; 3 University Lyon 1, F-69100 Villeurbanne, France

**Keywords:** transcranial random noise stimulation, auditory hallucinations, schizophrenia, transcranial direct current stimulation

## Abstract

Transcranial electrical stimulation has been proposed as a noninvasive therapeutic approach for reducing treatment-resistant symptoms of schizophrenia—in particular, auditory hallucinations. However, the high variability observed in the clinical response leaves much room to optimize the stimulation parameters and strengthen its benefits. We proposed to investigate the effects of high-frequency transcranial random noise stimulation (hf-tRNS), which is supposed to induce larger effects than conventional direct current stimulation. Here, we present an initial case series of ten patients with schizophrenia who underwent 10 sessions of 20 min hf-tRNS (2 mA, 100–500 Hz, 1 mA offset), with the anode placed over the left dorsolateral prefrontal cortex and the cathode over the left temporoparietal junction. Patients showed a significant reduction in auditory hallucinations after the hf-tRNS sessions (−36.1 +/− 21.8%, *p* = 0.0059). In this preliminary, open-label study conducted in ten patients with treatment-resistant symptoms of schizophrenia, frontotemporal hf-tRNS was shown to induce a substantial improvement in auditory hallucinations. Additional sham-controlled studies are needed to further evaluate hf-tRNS as a treatment for schizophrenia.

## 1. Introduction

Schizophrenia is a severe psychiatric disorder that is characterized by a variety of symptoms, traditionally divided into positive (e.g., delusions and hallucinations) and negative dimensions (e.g., apathy, lack of motivation, blunted affect). First-line treatments of schizophrenia rely on the use of antipsychotic medications. However, around 20–40% of patients have a poor or partial response to antipsychotics [1,2] and experience persistent symptoms such as auditory hallucinations. The lack of response to antipsychotics is associated with greater disability and impaired psychosocial functioning [3] and an increased risk of suicide attempts, which emphasizes the need to develop appropriate adjunctive therapeutic strategies to manage treatment-resistant symptoms of schizophrenia.

In the last 10 years, transcranial direct current stimulation (tDCS) has been proposed as a promising tool to reduce treatment-resistant symptoms of schizophrenia, with a focus on auditory hallucinations. tDCS is a noninvasive neuromodulation technique that consists of delivering a constant low-intensity direct current to the brain through a pair of electrodes placed on the scalp. The current that flows between the two electrodes is thought to modulate brain activity in a polarity-dependent manner by depolarizing or hyperpolarizing the membrane of the underlying neurons: currents entering the brain at the anode increase cortical excitability, whereas currents exiting the brain at the cathode decrease cortical excitability [4]. tDCS protocols for schizophrenia targeting auditory hallucinations have primarily used a “frontotemporal electrode montage”, with the anode placed over the left prefrontal cortex and the cathode over the left temporoparietal junction [5]. This montage was initially proposed based on the neurophysiological polarity-dependent properties of tDCS electrodes and neuroimaging studies demonstrating hyperactivity within temporoparietal areas and abnormal frontotemporal connectivity in patients with hallucinations [6,7]. Clinical studies reported that repeated sessions of frontotemporal tDCS can lead to significant beneficial effects on auditory hallucinations [5] (for a review, see [8]), but also on treatment-resistant negative symptoms [9]. However, despite promising results, some studies have failed to find significant clinical effects of frontotemporal tDCS on treatment-resistant symptoms of schizophrenia [10], and there are no clear, evidence-based consensus guidelines on the usefulness of tDCS for patients with schizophrenia [11,12].

In this context, stimulation with an alternative form of current, transcranial random noise stimulation (tRNS), has been proposed as a strategy to optimize the stimulation parameters in order to improve the clinical response. tRNS consists in delivering a current oscillating at random frequencies within a defined frequency range (<100 Hz for low-frequency tRNS and >100 Hz for high-frequency—hf-tRNS) and intensities. The current can be alternating, with electrodes continuously changing polarity, or unidirectional, where tRNS is delivered with a direct current (DC) offset [13,14]. tRNS has been shown to induce greater effects on cortical excitability than tDCS [15,16], and even more so when used with a DC offset [14] (see [17] for similar results on the superiority of tRNS with a DC offset on behavioral performance). Although the exact mechanisms by which hf-tRNS modulates brain activity in living humans remain unclear, tRNS is supposed to induce robust changes in cortical excitability by adding noise in the system, which can modulate the signal-to-noise ratio through stochastic resonance and make neural signal transmission and processing within neuronal populations more efficient [18]. tRNS with a DC offset is speculated to combine these characteristics of tRNS to add noise to the system to those of tDCS with polarization of the resting membrane potential.

The use of tRNS in the treatment of schizophrenia is attracting growing attention, as evidenced by the recent publication of two case reports and a recent pilot randomized controlled study (RCT) [19,20,21]. Two main electrode montages were used: hf-tRNS with DC offset showed beneficial effects on negative symptoms of schizophrenia when administered to the left dorsolateral prefrontal cortex [19,21], and positive symptoms and insight when administered using a left frontotemporal montage [20].

Here, we present an initial case series of patients with schizophrenia and auditory hallucinations who underwent 10 sessions of frontotemporal hf-tRNS with a DC offset. We hypothesized that applying hf-tRNS with such a left frontotemporal montage may lead to beneficial clinical effects on treatment-resistant auditory hallucinations in schizophrenia.

## 2. Materials and Methods

Ten patients with schizophrenia according to the DSM-5 criteria (6 women, 4 men) and presenting with severe daily treatment-resistant auditory hallucinations were recruited from our drug-resistant symptom management clinical unit at the Vinatier Hospital (Bron, France) and were addressed for treatment with noninvasive brain stimulation. Treatment resistance was defined as the presence of daily auditory hallucinations without remission despite taking antipsychotic medications at an optimized dosage for at least 6 weeks and after failure of at least one previous treatment with a different class of antipsychotics at an adequate dose and duration. One of the patients was not taking medication due to serious adverse drug reactions but had daily auditory hallucinations that persisted despite several trials and combinations of several classes of medication, including antipsychotics, in the past. All patients provided their informed consent. Demographic and clinical details of the patients are reported in Table 1. The study was a pilot study for an RCT registered on ClinicalTrials.gov NCT02744989 on 20 April 2016 and approved by a local ethics committee (CPP Sud Est VI, Ref AU 1231) on 8 January 2016, ANSM French Drug Regulatory Authority (Ref-ID-CRB: 2015-A01299-40).

**Figure 1:**
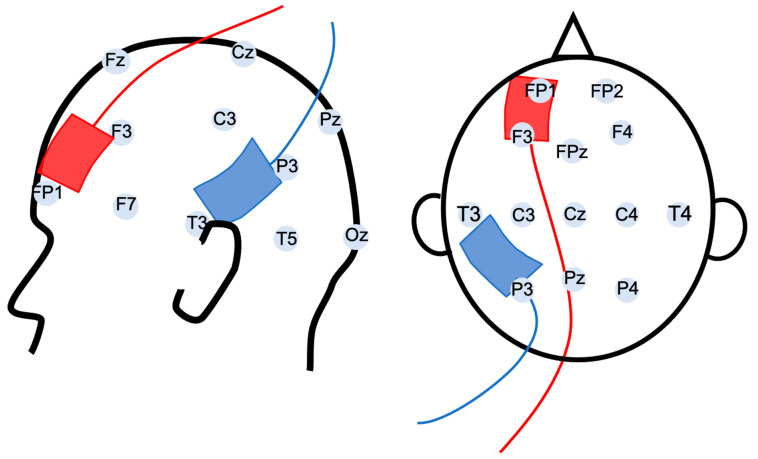
Illustration of the hf-tRNS electrodes montage with the anode (red) over the left prefrontal cortex (F3–FP1) and the cathode (blue) over the left temporoparietal junction (T3–P3) according to the 10/20 EEG international system of electrode placement.

Patients received 10 sessions (2 sessions per day over 5 consecutive days, separated by at least 2 h) of 20 min of hf-tRNS (100–640 Hz, 1 mA offset, 2 mA peak-to-peak, ramp up/ramp down of 30 s). hf-tRNS was delivered with a DC-Stimulator-Plus device (NeuroConn, Ilmenau, Germany), using two 7 × 5 cm (35 cm^2^) electrodes placed in sponges soaked in a saline solution (0.9% NaCl). The electrodes were placed according to a left frontotemporal montage, with the anode placed with the middle of the electrode over a point midway between F3 and FP1 and the cathode over a point midway between T3 and P3 (according to the international 10–20 electrode placement system, Figure 1).

The severity of auditory hallucinations was assessed with the Auditory Hallucination Rating Scale (AHRS) [22,23] and the severity of symptoms of schizophrenia with the Positive and Negative Syndrome Scale (PANSS). Symptom severity was assessed twice by a trained psychiatrist, once at baseline and again within 3 days of the last hf-tRNS session.

The AHRS and PANSS scores (total PANSS score and scores for each subscale of the PANSS: positive, negative and general psychopathology) at baseline and after hf-tRNS were compared using Wilcoxon signed-rank tests. Additionally, the effect sizes *r* were calculated as Z statistics divided by the square root of the sample size. The interpretation values for r according to Cohen’s classification are 0.1 to 0.3 (small effect), 0.3 to 0.5 (moderate effect) and superior to 0.5 (large effect).

## 3. Results

All the patients received 10 sessions of hf-tRNS. Stimulation sessions were well tolerated by all patients and no adverse effects were observed. The mean age of the patients was 39.9 years (*SD* = 14.2, range 22–67). Patients’ medication was maintained unchanged throughout the study period (Table 1).

### 3.1. Effects on Auditory Hallucinations

**Figure 2:**
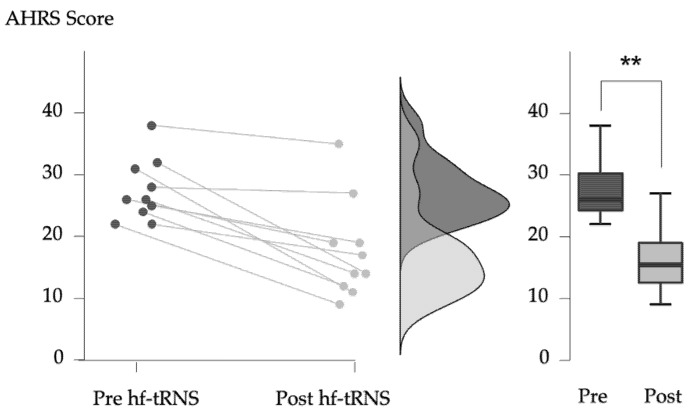
Individual effects of 10 sessions of frontotemporal hf-tRNS on auditory hallucinations measured with the AHRS (Auditory Hallucination Rating Scale). ** indicates statistically significant difference (*p* < 0.01).

After the 10 sessions of hf-tRNS, the patients showed a significant reduction in auditory hallucinations from a mean AHRS score of 27.40 ± 5.02 (standard deviation) to 17.70 ± 7.96 (mean reduction of −36.1% ± 21.8, *W* = 55.0, *p* = 0.0059, Figure 2), corresponding to a large effect size (*r* = 0.89).

Regarding treatment response, 40% of patients showed at least a 50% diminution in auditory hallucinations (Patients #5, 6, 7 and 9) and 80% of patients achieved at least a 20% diminution in hallucinations (Patients #1, 2, 5, 6, 7, 8, 9 and 10). Among the non-responders (Patients #3 and 4), there was one young drug-free female patient and one young female who was treated with clozapine and levomepromazine.

### 3.2. Effects on Other Symptoms of Schizophrenia

Due to lost or missing data, pre- and post-hf-tRNS PANSS scores were not available for all 10 participants. Mean reductions in PANSS scores were observed after hf-tRNS (−16.4 ± 20.1%, *n* = 6, W = 18, *p* = 0.140). Only reductions in the positive subscale of the PANSS reached significance (−13.6 ± 4.5%, *n* = 7, *W* = 28, *p* = 0.020, *r* = 0.91), while reductions in the negative subscale (−14.2 ± 30.6%, *n* = 7, *W* = 18, *p* = 0.142), general psychopathology subscale (−15.1 ± 22.8%, *n* = 6, *W* = 18, *p* = 0.156) and total PANSS scores were not significant (Table 2).

Regarding the response rate, with a response criterion defined as at least a 20% decrease in total PANSS scores [9], we observed that 50% of patients with available data (3 out of 6) could be classified as responders after the 10 hf-tRNS sessions (Patients #3, 5 and 6). It is interesting to note that the drug-free patient without a significant improvement in hallucinations (Patient #3) presented, however, a significant improvement in the global symptomatology.

## 4. Discussion

In this preliminary, open-label study conducted in 10 patients with schizophrenia experiencing treatment-resistant auditory hallucinations, we found a substantial beneficial effect of frontotemporal hf-tRNS on auditory hallucinations. The mean reduction in auditory hallucinations (−36.1% at the AHRS) observed after 10 sessions of hf-tRNS was similar to those obtained with conventional frontotemporal tDCS (around −30% in [5,24]). The use of hf-tRNS would therefore not provide an additional efficacy gain over tDCS for auditory hallucinations, as is the case for negative symptoms, where hf-tRNS applied to the left dorsolateral prefrontal cortex has been shown to improve negative symptoms with a larger effect size than conventional tDCS [21].

In the current study, all patients had auditory hallucinations resistant to antipsychotic treatments and the large majority of them (7 out of 10) were on clozapine. Therefore, hf-tRNS may be an appropriate approach in participants with high levels of antipsychotic resistance. This is of great importance, as other approaches with noninvasive brain stimulation techniques, such as repetitive transcranial magnetic stimulation (rTMS) at the left temporoparietal junction, do not seem to be effective in reducing auditory hallucinations in schizophrenia patients treated with clozapine [25], and the use of clozapine has been associated with decreased tDCS effects [9]. Although the place of hf-tRNS or tDCS versus rTMS in the treatment of schizophrenia has yet to be defined, hf-tRNS may therefore have advantages over rTMS in the specific case of resistance to clozapine, in addition to being a less expensive, more accessible and highly deployable tool that can be considered for home use in the future.

In addition to the decrease in auditory hallucinations, we also observed a −16.4% decrease in PANSS scores after hf-tRNS. Although not statistically significant, the observed decrease was comparable to the 13% decrease that we observed in an RCT using tDCS for daily treatment-resistant hallucinations [5] and to the results of a large RCT investigating the effect of tDCS on treatment-resistant negative symptoms of schizophrenia (−10.4%, [9]). This lack of significant effect can in part be explained by the timing of the evaluation. There was only one week between the baseline evaluation and the post hf-tRNS evaluation. A study with a longer follow-up period is warranted to investigate whether the beneficial effects observed acutely on hallucinations may translate into beneficial outcomes on the other symptoms of schizophrenia, particularly negative symptoms, and on functioning. Importantly, 50% of patients were classified as responders to hf-tRNS when response was defined as at least a 20% improvement in total PANSS scores. This is higher than in other studies investigating the effects of tDCS on schizophrenia symptoms [5,9]. However, these results should be taken with caution as the study was not specifically designed to assess PANSS scores, and, because of missing data, only six patients had available pre- and post-hf-tRNS PANSS scores. Although only the decrease in the positive subscale of the PANSS reached statistical significance, the observed improvements seemed to be similar in all subscales of the PANSS, suggesting an overall nonspecific impact of hf-tRNS on all dimensions of the disease, rather than an effect limited to the negative subscale, as reported in the literature [5,9].

In this preliminary study, we proposed to modify the current shape in a strategy to optimize the stimulation parameters to improve the clinical response. One of the other possible means of optimization could have been to increase the focality of stimulation by using High-Definition tDCS (HD-tDCS). Indeed, we used a conventional frontotemporal electrode montage with two large 35cm^2^ electrodes placed on two target sites, which induced current flows through large and widespread brain areas, not only the targeted ones. Modeling of the electric field distribution induced by such a frontotemporal tDCS montage even revealed that the highest current intensities fall outside the targeted brain regions [26], with a peak of current in the left central sulcus region and Broca’s area depending on individual characteristics [27]. In contrast, HD-tDCS has the potential for precise and focused stimulation and has shown longer-lasting effects than conventional tDCS [28,29,30]. This technique uses small, gel-based ring electrodes that can be flexibly arranged in an optimal montage to elicit a specific response in the targeted brain region and allows the simultaneous targeting of multiple brain regions instead of a single point target [31]. HD-tDCS has shown promising results in reducing auditory hallucinations in patients with schizophrenia in a case report [32] and an open-label study [33]. To our knowledge, there is no report in the literature of HD-hf tRNS in patients with schizophrenia.

This preliminary study suffers from several limitations, the first being the relatively small sample size. In addition, a placebo effect cannot be excluded from the current observations due to the lack of a control group and the absence of blinding. Further investigations with the RCT methodology are needed to allow a clear conclusion on the effects of hf-tRNS in a clinical setting, especially as tRNS offers more effective blinding than other electrical stimulation techniques, including tDCS [34]. Furthermore, only the acute effect of hf-tRNS was measured, within 3 days after the last stimulation session. Future studies should examine whether the observed beneficial effects can be maintained over time, which is of crucial importance for patients. For example, in previous studies on the clinical effects of tDCS on auditory hallucinations and negative symptoms, a sustained beneficial effect after 3 months was observed [5,9]. Nevertheless, this study, with its promising preliminary effects, paves the way for the deployment of a large RCT investigating the acute and long-term effects of active hf-tRNS compared to sham in patients with schizophrenia [35].

In conclusion, the present study suggests beneficial effects of 10 sessions of frontotemporal hf-tRNS (two sessions per day for 5 days) on auditory hallucinations in patients with schizophrenia resistant to antipsychotic treatments, including clozapine. Our results support the development of large-scale RCTs to further evaluate the usefulness of hf-tRNS as a therapeutic tool for the treatment of auditory hallucinations in patients with schizophrenia and how the improvement in hallucinations may translate into beneficial outcomes for other symptoms of schizophrenia and patients’ functioning.

## Figures and Tables

**Table 1:** Sociodemographic and clinical characteristics of patients at inclusion.

Patient	Sex	Age	AHRS	PANSS	Antipsychotic Medication
1	F	67	26	62	olanzapine LP 300 mg/15d + amisulpride 800 mg/d
2	M	44	26	43	clozapine 500 mg/d
3	F	26	38	91	drug free
4	F	38	28	70	clozapine 600 mg/d + levomepromazine 100 mg/d
5	M	27	32	83	clozapine 500 mg/d + chlorpromazine 200 mg/d + risperidone 6 mg/d
6	F	38	22	66	clozapine 300 mg/d + cyamemazine 150 mg/d
7	M	42	31	NA	clozapine 400 mg/d + aripiprazole 15 mg/d
8	F	22	22	NA	aripiprazole 25 mg/d
9	M	59	24	68	olanzapine 30 mg/d + clozapine 25 mg/d
10	F	36	25	83	clozapine 600 mg/d

Age, in years; AHRS, auditory hallucination rating scale; d, day; NA, not available (missing data); PANSS, Positive and Negative Syndrome Scale; F, female; M, male.

**Table 2:** Evolution of clinical scores after 10 sessions of hf-tRNS. Results are given as mean (standard deviation).

	*n*	Pre-hf-tRNS	Post-hf-tRNS	*p*
PANSS total score	6	69.17 (16.8)	56.33 (13.4)	0.140
Positive symptoms	7	16.86 (4.10)	14.57 (3.51)	0.020
Negative symptoms	7	19.00 (4.97)	15.57 (4.89)	0.142
General psychopathology	6	34.33 (10.05)	27.67 (6.41)	0.156

## Data Availability

The data presented in this study are available on request from the corresponding author.
